# Using the photon isoeffective dose formalism to compare and combine BNCT and CIRT in a head and neck tumour

**DOI:** 10.1038/s41598-023-50522-5

**Published:** 2024-01-03

**Authors:** Ian Postuma, Chiara Magni, Barbara Marcaccio, Setareh Fatemi, Valerio Vercesi, Mario Ciocca, Giuseppe Magro, Ester Orlandi, Barbara Vischioni, Sara Ronchi, Yuan-Hao Liu, Yang Han, Changran Geng, Sara Josefina González, Silva Bortolussi

**Affiliations:** 1 National Institute of Nuclear Physics, INFN, Unit of Pavia, Pavia, 27100 Italy; 2 Department of Physics, University of Pavia, Pavia, 27100 Italy; 3 National University of San Martín, Dan Beninson Institute, Buenos Aires, Argentina; 4 National Centre for Oncological Hadrontherapy, CNAO, Pavia, 27100 Italy; 5 Neuboron Medtech Ltd, Nanjing, China; 6 Department of Nuclear Science and Technology, Nanjing University of Aeronautics and Astronautics, NUAA, Nanjing, China; 7 National Atomic Energy Commission, CNEA, Buenos Aires, Argentina; 8 National Scientific and Technical Research Council, CONICET, Buenos Aires, Argentina

**Keywords:** Radiotherapy, Cancer

## Abstract

Boron Neutron Capture Therapy (BNCT) is a radiotherapy technique based on the enrichment of tumour cells with suitable 10-boron concentration and on subsequent neutron irradiation. Low-energy neutron irradiation produces a localized deposition of radiation dose caused by boron neutron capture reactions. Boron is vehiculated into tumour cells via proper borated formulations, able to accumulate in the malignancy more than in normal tissues. The neutron capture releases two high-LET charged particles (i.e., an alpha particle and a lithium ion), losing their energy in a distance comparable to the average dimension of one cell. Thus BNCT is selective at the cell level and characterized by high biological effectiveness. As the radiation field is due to the interaction of neutrons with the components of biological tissues and with boron, the dosimetry requires a formalism to express the absorbed dose into photon-equivalent units. This work analyzes a clinical case of an adenoid cystic carcinoma treated with carbon-ion radiotherapy (CIRT), located close to optic nerve and deep-seated as a practical example of how to apply the formalism of BNCT photon isoeffective dose and how to evaluate the BNCT dose distribution against CIRT. The example allows presenting different dosimetrical and radiobiological quantities and drawing conclusions on the potential of BNCT stemming on the clinical result of the CIRT. The patient received CIRT with a dose constraint on the optic nerve, affecting the peripheral part of the Planning Target Volume (PTV). After the treatment, the tumour recurred in this low-dose region. BNCT was simulated for the primary tumour, with the goal to calculate the dose distribution in isoeffective units and a Tumour Control Probability (TCP) to be compared with the one of the original treatment. BNCT was then evaluated for the recurrence in the underdosed region which was not optimally covered by charged particles due to the proximity of the optic nerve. Finally, a combined treatment consisting in BNCT and carbon ion therapy was considered to show the consistency and the potential of the model. For the primary tumour, the photon isoeffective dose distribution due to BNCT was evaluated and the resulted TCP was higher than that obtained for the CIRT. The formalism produced values that are consistent with those of carbon-ion. For the recurrence, BNCT dosimetry produces a similar TCP than that of primary tumour. A combined treatment was finally simulated, showing a TCP comparable to the BNCT-alone with overall dosimetric advantage in the most peripheral parts of the treatment volume. Isoeffective dose formalism is a robust tool to analyze BNCT dosimetry and to compare it with the photon-equivalent dose calculated for carbon-ion treatment. This study introduces for the first time the possibility to combine the dosimetry obtained by two different treatment modalities, showing the potential of exploiting the cellular targeting of BNCT combined with the precision of charged particles in delivering an homogeneous dose distribution in deep-seated tumours.

## Introduction

Boron Neutron Capture Therapy (BNCT) exploits the thermal neutron capture in $^{10}$B to deliver a selective dose to tumour. It consists in the administration of a borated drug and in the subsequent irradiation with a neutron beam having suitable spectral characteristics^1^. The neutron capture reaction in $^{10}$B generates two high-LET, short-range charged particles that lose all their energy inside or in proximity of the cell where the reaction occurs. Thus, provided a sufficient tumour-to-normal tissue $^{10}$B concentration ratio, the neutron irradiation produces lethal damages to the tumour with a substantial limitation of the dose absorbed by surrounding healthy tissues. The availability of neutron beams obtained with proton accelerators is giving a boost to BNCT clinical application, also promoting the exploration of new modalities such as the combination of BNCT and other forms of hadrontherapy. BNCT main characteristic is its intrinsic selectivity, potentially useful to treat infiltrated and disseminated malignancies. Dose is prescribed to the maximum tolerable dose of the most radiosensitive healthy tissue involved in the irradiation; the differential boron concentration allows the deposition of a higher dose in the tumour. The dosimetry is calculated via Monte Carlo simulation, knowing boron concentration in the tissues. Charged particle therapy, on the other side, exploits the Bragg peak to generate a high conformal dose deposition in the tumour. The possibility to combine BNCT and charged particle treatment could allow a more uniform and precise dose administration to the tumour bulk and the targeting of the tumour in proximity of sensitive organs or in areas where tumour cells could be infiltrated but not yet detectable. In order to combine dose distributions due to different types of particle therapy, it is mandatory to express the dose values in a common language, i.e. photon-equivalent units. Traditionally, BNCT photon-equivalent dose has been calculated with fixed Relative Biological Effectiveness factors (RBE) and Compound Biological Effectiveness factors (CBE) each multiplying the 4 components of BNCT mixed-field radiation field^2^. This approach had already been questioned in 2001 by Coderre, who had first proposed it, because dosimetry was not able to explain the radiobiological results in the healthy brain of dogs and patients receiving BNCT at the Brookhaven Medical Research Reactor, compared to expected responses to photon irradiation. The findings indicated that RBE-weighted doses in brain might be underestimated, while doses to tumor could be significantly overestimated^3^. Recently, the fixed RBE model has been proved not suitable, for being formally incorrect and for lack of prediction when analyzing the clinical outcome of BNCT patients, compared with photon therapy^4–6^. The dose calculated in tumour are in fact artificially high and fail to describe a consistent dose-clinical effect relation in retrospective studies. Moreover, when used to calculate a Normal Tissue Complication Probability in 6 patients treated in Finland, RBE-weighted dose led to inconsistent results^5^; ongoing work is confirming this fact for a larger number of patients. A more refined model to translate BNCT dose into photon equivalent units is the photon isoeffective dose formalism, that takes as input in vitro or in vivo radiobiological experiments and includes the whole information of the curves obtained with BNCT, neutron-only and photon irradiation of different biological models^4,5,7,8^. This approach was proved more consistent in describing dosimetry and predicting clinical results in BNCT patients, both regarding tumour control and normal tissue complications for different types of tumours and healthy tissues: brain^8^, mucosa^5^, head and neck tumours^6^, osteosarcoma^9^. Head and neck cancer has been one of the most investigated targets of BNCT so far, with clinical trials in Japan, Taiwan and in Finland. In Argentina veterinary treatments have been carried out in canine patients^10^. In Helsinki, Finland, more than 100 patients received BNCT at the reactor of VTT Technical Research Centre of Finland between 2003 and 2012. For 69 cases of squamous cell carcinoma, the 2-year locoregional progression-free survival rate was 38% and the overall survival rate 21%^11^. In Japan, patients with either locoregional recurrence or newly diagnosed head and neck cancer were treated at KURRI (Kyoto) between 2001 and 2007, resulting in overall response rate of 58% within 6 months and median survival time of 10.1 months after treatment^12^. In Taiwan, 17 patients with 23 recurrent head and neck were treated with with a 2-year Overall Survival (OS) of 47%. A second trial was performed combining BNCT and Intensity Modulated Radiotherapy^13^. Recently, the results of a phase II trial using the C-Bens accelerator in Japan have been reported. Accelerator-based BNCT produced tumour response in 71% of head and neck patients, with no serious adverse events^14^.

Carbon-ion radiotherapy (CIRT) is confirmed to be a valid option for unresectable and recurrent radioresistant head and neck cancer, adenoid cystic carcinoma (ACC) particularly^15,16^. Today, more than 1500 treatments of the head and neck tumours were carried out with active scanning carbon ion at the Italian National Centre for Oncological Hadrontherapy (CNAO) in Pavia, Italy (http://fondazionecnao.it)^17^. However, in some cases, the malignancy may be too close to very radiosensitive tissues/organs so that a proper dose coverage of the Planning Target Volume (PTV) may not be optimal even with highly biological effective charged particle beams.

In this work, we show how photon isoeffective dose model can be used to simulate the BNCT dosimetry of a patient with a locally advanced unresectable ACC with macroscopic perineural invasion that was treated with CIRT at CNAO. This clinical case, for which the CIRT dosimetry and the medical history were known, was taken as a benchmark. Tumour Control Probability (TCP) was used as a figure of merit to compare the dosimetry distribution with a simple criterion, that can be straightforwardly related to the clinical outcome of the patient. At three-year follow-up after CIRT, the patient was diagnosed with a tumour in a region surrounding the optic nerve, which was not treated with CIRT. As the tumour could not be controlled with the dose distribution achieved with CIRT, BNCT has been explored to study three possible scenarios with these goals:

1. Evaluation of BNCT for the primary tumour to understand the potential of the isoeffective formalism in predicting a TCP consistent with that of CIRT, for comparison purpose.
2. Evaluation of BNCT as a potential treatment for the recurrence, for which the cellular selectivity may deposit a therapeutic dose in tumour while sparing the optic nerve. The TCP due to BNCT can be compared to that of the primary tumour as a figure of merit to understand the therapeutic potential of BNCT for the recurrence.
3. Evaluation of the combination of carbon ion irradiation and BNCT, towards the possibility to conceive this kind of treatment for complex tumours, to explore a potential synergy between BNCT selectivity and carbon ions precision.

This study evaluates the inter-comparison and the possible CIRT and BNCT sequential application from a dosimetric point of view. It aims at demonstrating that the computational models developed so far are an appropriate tool to address this task.

## Methods

### BNCT photon isoeffective dose

BNCT dose is due to a mixed field, with different dose components: protons from $^{14}$N(n,p)$^{14}$C reaction and from neutron scattering on hydrogen; $\alpha$ and $^7$Li from neutron capture in $^{10}$B; photons, from radiative capture in hydrogen and from the gamma background in the neutron field. The photon isoeffective dose is the dose of the reference photon radiation which causes the same effect as the combination of the four dose components of the BNCT mixed-field^4^. If *in vivo* data are available, the effect used to compare BNCT and the reference radiation can be a relevant radiobiological figure of merit, such as TCP^5^. For head and neck cancer, the parameters of the model were obtained from the tumour control rate of large number of lesions (about 480) irradiated using photons and BNCT (with and without the infusion of the L-boronophenylalanine fructose boron compound). The radiobiological data used to construct the model were collected from: photon irradiation (R)^5^, neutron beam only irradiation (BO)^5^, neutron irradiation in the presence of the boron compound L-boronphenylalanine fructose or L-BPA-F (BPA-BNCT)^18–22^. The animal model was optimized to be representative for the study of effects in tumour and toxicity in normal tissues as described in^5^. A TCP model was obtained and used in the determination of the photon isoeffective dose: the dose of the reference radiation leading to the same TCP as the combination of the four BNCT radiation components^5^. The photon isoeffective dose for brain and optic nerve was derived from the procedure presented in Pedrosa-Rivera et al.^8^, that uses the RBE and CBE factors previously determined by Morris et al.,^23^, from the radiobiological data published in^23,24^, on the myelopathy effects on the spinal cord of rats (endpoint 50% incidence) irradiated with a BNCT and photon beam. Dose-response curves for photons and BNCT (with and without the infusion of the L-BPA-F) comprised about 300 animals.

CIRT plan is given in photon-equivalent units, converting absorbed dose using the Local Effect Model (LEM)^25^.

To be consistent with the IAEA & ICRU recommendations on the dose reporting^26^, photon-equivalent dose values are reported with the notation Gy (IsoE). For clarity, carbon-ion doses are defined in Gy (IsoE; C12) and BNCT in Gy (IsoE; BNCT).

For a fair comparison between CIRT and BNCT, we translated carbon ion dose values into single fraction doses using BED formalism^27^, since BNCT is usually delivered as a single fraction treatment. The conversion was obtained considering alpha/beta equal to 2 Gy consistently to what assumed in the clinical practice for the LEM calculation. In this case, the notation is Gy (IsoE; C12; BED).

### Calculation of tumour control probability (TCP)

A tumour control probability model for non-uniform single-fraction photon doses was derived for head and neck cancer in^6^. This model was selected for TCP calculations in this work. Two characteristics make the application of such a model a suitable and an interesting option for the proposed analysis. First, the TCP model was constructed based on the dose, volume and rate of complete response for about 100 head and neck cancer patients who underwent stereotactic body radiotherapy (SBRT)^28^. Briefly, fractionated doses were first converted to single-fraction (SF) values using the linear-quadratic formalism for an alpha/beta ratio of 10 Gy. Then, assuming that the tumor control probability for a SF uniform photon dose *D* is correctly described by

1 $$\begin{aligned} TCP(v,D)=e^{-c_1v^{c_2}S(D)}, \end{aligned}$$

with *v* the tumor volume (in cm$^3$), $c_1$ and $c_2$ the parameters modulating its effect on the probability of local control, and *S*(*D*)=exp(-$\alpha$ $D$-$\beta$ $D^2$) the probability of cell survival, the model parameters were obtained by fitting Eq. (1) to the data. Thus, the adjusted model parameters with the 68% Confidence Interval ($CI_{68\%}$), $c_1$ = 2.4 [1.1, 3.7], $c_2$ = 0.12 [0, 0.26], $\alpha$ = 0.022 [0.016, 0.028], were derived from a true-to-life clinical scenario. Second, the TCP model takes into account non-uniform dose distributions by applying the equivalent subvolume model reported in^29^ to Eq. (1). It follows that the TCP for the tumor *T* can be expressed as:

2 $$\begin{aligned} TCP_T=e^{-c_1v^{c_2}(\int _{T} S(D({\textbf{x}}))^{1/c_2}\frac{dx}{v})^{c_2}} \end{aligned}$$

where $D({\textbf{x}})$ denotes the spatial dose in a subvolume of *T* around the point *x*. Due to the typical target size of head and neck cancers, which is large if compared to the neutron beam penetration, significant dose inhomogeneity could be observed in a BNCT treatment.

For the calculation of the photon isoeffective dose and the analysis of the results, we employed the IT_STARTS toolkit (developed at the National Institute of Nuclear Physics, Unit of Pavia, in collaboration with the National Atomic Energy Commission of Argentina) consisting in an open-source program based on Python™^30^ and Python packages^31–35^. The toolkit works as a dose-engine, converting the calculated dose using user-specified radiobiological models and superimposing the isodose curves on the medical images of the patient. Moreover, it allows building the Dose Volume Histograms (DVH) for a certain Region of Interest (ROI) and calculating radiobiological figures of merit, such as the TCP.

### Clinical case

The case study is a patient affected by an ACC arising from the minor salivary glands of the masticatory space with the involvement of perineural pathways. All medical data were anonymous and shared between Institutions in accordance with relevant guidelines and regulations holding at the moment of the research here described.

**Figure 1:**
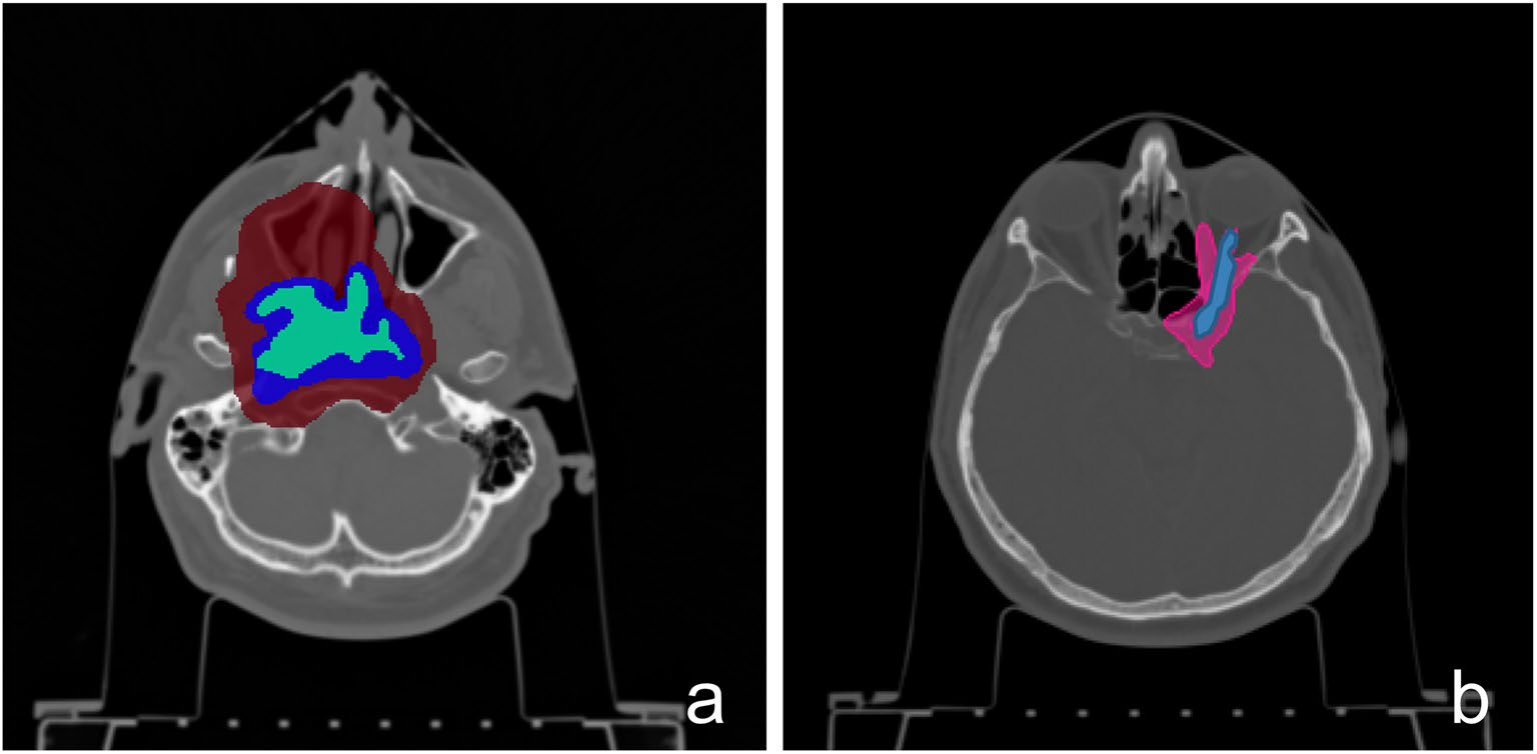
CT image of the patient: (**a**) with the primary tumour (in red the PTV-LD, in blue the PTV-HD and in green the GTV), and (**b**) with the recurrence in pink and the optic nerve in blue.

The gross target volume (GTV) was 57.4 cm$^3$. The Planning Target Volume (PTV) was divided into two parts: low-dose PTV (PTV-LD) and high-dose PTV (PTV-HD), as shown in Fig. 1a.

A total dose of 68.8 Gy (IsoE;C12) was administered to the PTV in 16 consecutive fractions, divided as follows: 9 fractions to the PTV-LD (38.7 Gy (IsoE;C12)), including the PTV-HD volume, and 7 additional fractions to the PTV-HD (30.1 Gy (IsoE;C12)). Figure 1a shows PTV-LD, the PTV-HD and the GTV for the case under study. The dimension of the PTV-LD and the location of the GTV make BNCT considerably challenging, from the point of view of neutron penetration. However, it is worth noting that patients who received BNCT in the above-mentioned trials were also affected by large tumours. For example, in Finland, the median GTV volume was 105 cm$^3$, ranging from 53 to 187 cm$^3$^11^. The primary tumour of the chosen clinical case is thus a representative example of a realistic BNCT candidate.

Table 1 reports CIRT dose delivered with the fractionated scheme for PTV-LD, PTV-HD and GTV as well as doses for relevant healthy organs.

**Table 1:** Dose values for PTV, GTV and healthy tissues of the original treatment planning with carbon-ion beam.

ROI		Total dose given in fractioned scheme		
Name	Volume	[Gy (IsoE; C12)]		
	[cm$^3$]	Min	Mean	Max
PTV-LD	263.5	16.0	61.3	83.9
PTV-HD	102.9	31.6	70.9	81.8
GTV	57.4	43.6	71.8	81.8
Brain	1235	0	4.5	32.0
Right optic nerve	1.4	0.3	13.1	32.3
Left optic nerve	1.2	13.8	21.0	28.2

As anticipated above, the tumour relapsed after three years, in a region surrounding the optic nerve. Figure 1b shows the tumour in pink and the optic nerve in blue.

### BNCT treatment planning for GTV and recurrence

The Treatment Planning System employed is NCTPlan^36^, which reads a stack of medical images and produces a voxelized model of the patient, written as an input for the Monte Carlo code MCNP^37^. It also allows establishing the beam directions and writes the source positions in the input file. The spectral and geometrical characteristics of the source are then specified, and the simulation is run. Dose is calculated in 1 cm$^3$ voxels, assuming KERMA approximation.

Italian National Institute of Nuclear Physics (INFN) designed and built a radiofrequency quadrupole proton accelerator (RFQ) able to deliver a high-flux neutron beam by interaction of 5 MeV-30 mA proton with a thin beryllium target. A Beam Shaping Assembly (BSA), whose core is made by lithiated aluminium fluoride, allows obtaining an epithermal neutron beam suitable for BNCT of deep-seated tumours^1^. This beam proved to be as effective as the one at the Finnish reactor-based facility, where several head and neck cancer patients safely received BNCT^38,39^, and thus it was used for treatment planning simulations in this study.

The BNCT treatment was simulated following the protocol adopted in Finland consisting in two applications, using two beam ports each time. The median time between the treatments was approximately 6 weeks. Boron concentration was also taken from the Finnish clinical experience in head and neck cancer, with administration of 350 mg of BPA per kg of body weigh, two hours before the irradiation. Table 2 lists the boron concentration values assumed in different tissues.

**Table 2:** $^{10}B$ concentration values assumed in tissues^40^.

Tissue	$^{10}B$ concentration [microg/g]
Brain, optic nerve, eye	15
skin	22.5
mucosa	30
tumour	52.5

The prescription is to the maximum dose to mucosa, which absorbs about twice the concentration of boron than other normal tissues. The adverse effect limiting the treatment is the oral mucositis grade 3 or higher caused by the depletion of mucosa cells^41^. All the normal tissues outside the organs contoured in the original CIRT were precautionary as mucosa, to work with the worst case scenario. Following the Finnish protocol, the treatment was limited to deliver a maximum absorbed dose of 6 Gy in mucosa in each BNCT application. The maximum dose delivered to eyes, optic nerve and skin and the mean dose delivered to the brain was also calculated and compared to the limiting values^42^ proving that the criterion adopted to protect mucosa was the most conservative. This turns into a very conservative criterion to limit the irradiation; in fact, the maximum dose rate may occur in a more radio-resistant tissue as muscle, for example. However, it is preferable to adopt a more cautious approach to protect the patient from adverse effects.

**Figure 2:**
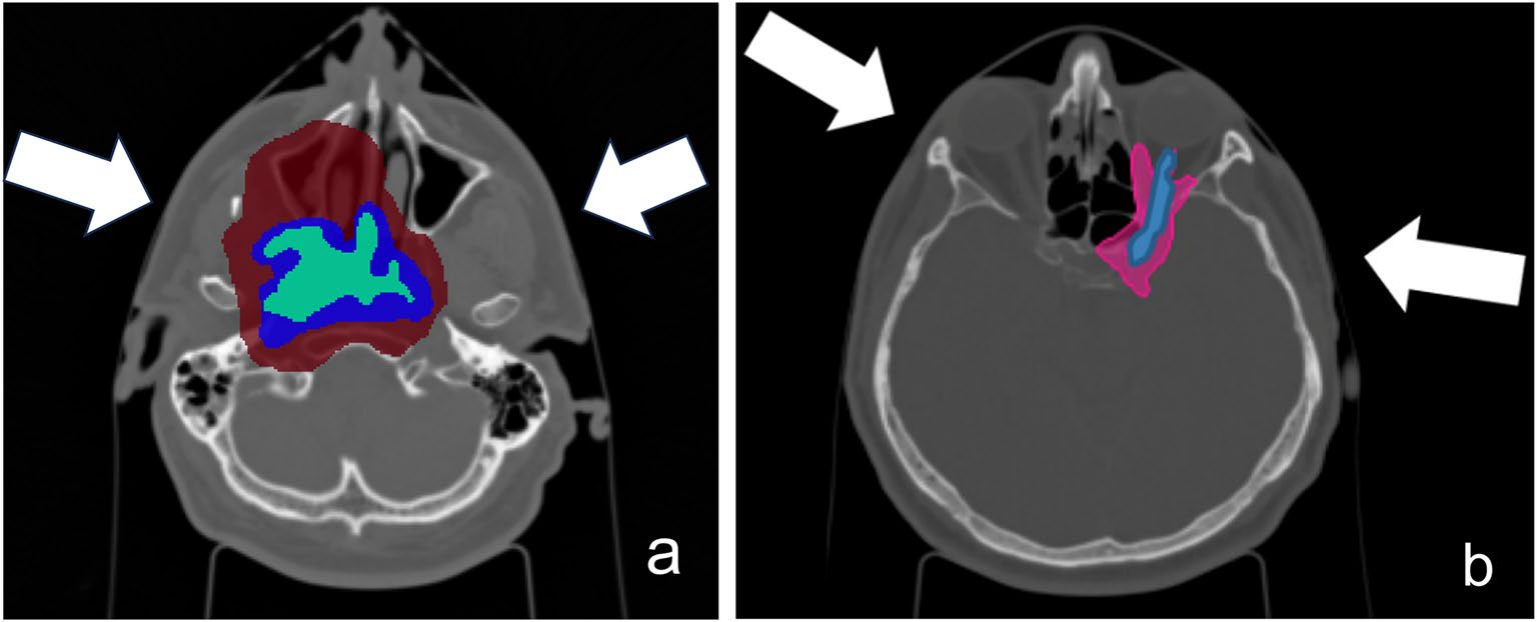
Position of the beams for the irradiation of the primary tumour (**a**) and of the recurrence (**b**). The white arrows represent the neutron beam-port positions for the two BNCT applications.

The beam orientation was optimized to maximize the dose to GTV while sparing the normal tissues (Fig. 2a).

For the recurrence, the same parameters and strategies were adopted as before and the beams orientation is shown in Fig. 2b. The photon isoeffective dose in optic nerve was verified to be lower than tolerance level when a maximum of 6 Gy was prescribed to the mucosa.

### Combination of CIRT and BNCT

To simulate a combined treatment, we considered a single BNCT irradiation coupled to CIRT of the PTV-LD or PTV-HD: one session of BNCT added to the 9 fractions of CIRT covering the PTV-LD or one session of BNCT added to the 7 fractions of CIRT covering the PTV-HD. We thus obtained a dose distribution which is the sum of the photon isoeffective doses of the two treatments using the BED formalism. As for the BNCT treatment, we considered that healthy tissue completely recovers between the carbon-ion irradiation and BNCT (as occurs between the two sessions of BNCT in the Finnish protocol). Therefore, the BNCT treatment was once again planned by limiting the healthy tissue at risk (i.e. mucosa) to 6 Gy. The BED dose due to carbon-ion irradiation was kept as in the original treatment.

### Ethical approval

The approval to use the clinical and therapy data included in this manuscript has been granted by the Territorial Ethical Committee Lombardy 6 (protocol 004332/23).

## Results

### Primary tumour

**Figure 3:**
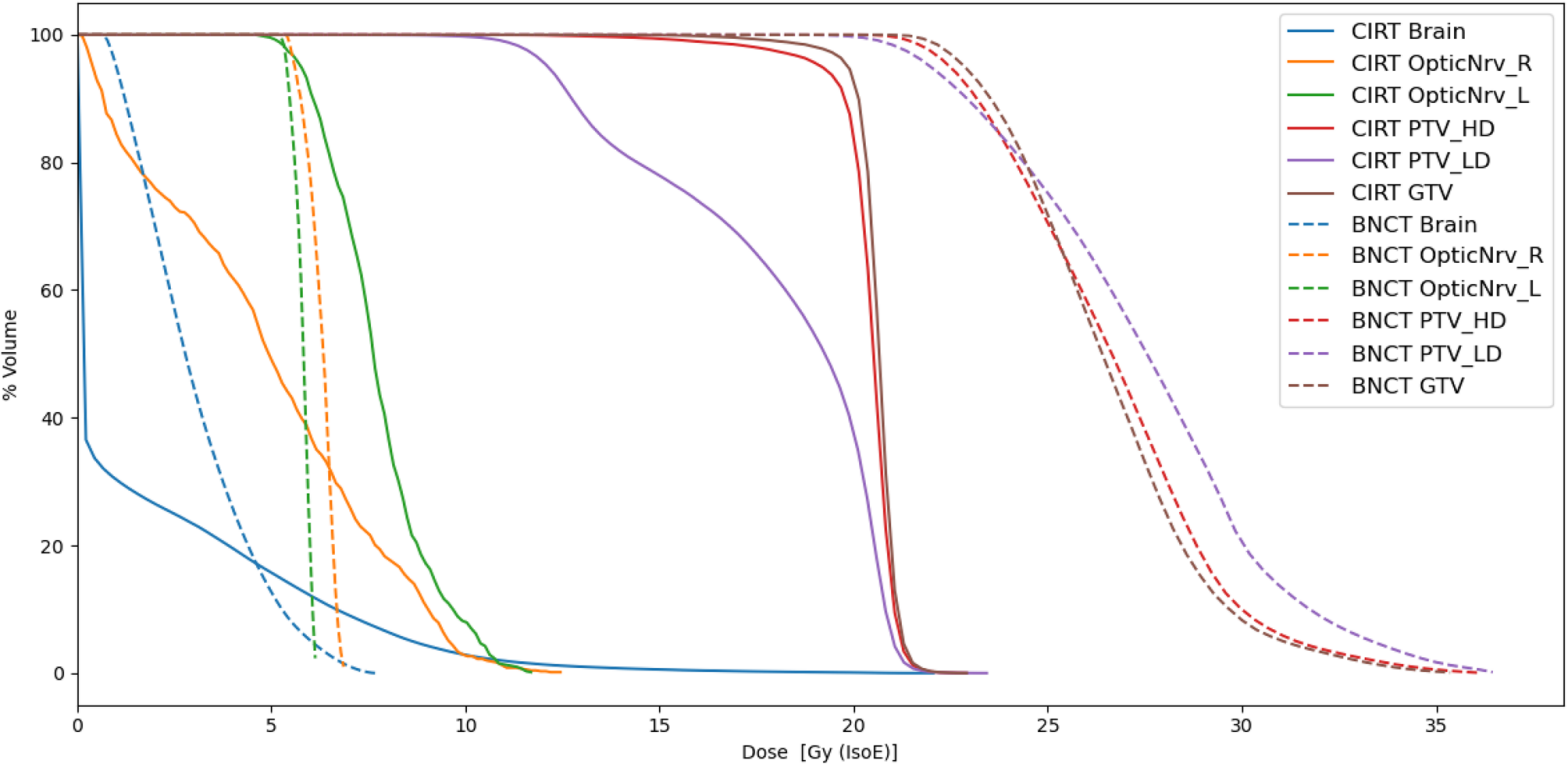
DVH of the CIRT (in single-fraction) and of the BNCT treatment. Gy (IsoE) of x-axis indicates Gy (IsoE, C12, BED) for CIRT and Gy (IsoE, BNCT) for BNCT.

The limitation of the dose delivered to oral mucosa led a total treatment time of 25.7 min for each of the two BNCT sessions. Figure 3 reports the Dose Volume Histograms (DVH) for carbon-ion plan converted into one fraction and for the two sessions of BNCT.

**Table 3:** Homogeneity index. PTV-LD has not been included because dose is not intended to be uniform in this volume.

	PTV-HD	GTV
BNCT	0.33	0.31
CIRT	0.17	0.11

The uniformity of dose distribution has been quantified with the homogeneity index (HI) (D$_2$ - D$_{98}$)/D$_{mean}$, where D$_x$ is the dose received by at least x% of the volume. Results for the three volumes of interest are listed in table 3.

As expected, BNCT dose is not as uniform as carbon-ion dose in the GTV, which is due to the size of the tumour combined with the characteristic thermal neutron field generated in depth by an epithermal neutron beam. Table 4 shows the BNCT and carbon-ion dose statistics for the target ROIs. Dose values have been translated into single-fraction using BED formalism for CIRT with an alpha/beta equal to 2 Gy. The BNCT dose values were translated into photon-equivalent units using isoeffective dose model^5^, which uses photon radiation therapy data reported in BED formalism with the same alpha/beta value. Values shows that BNCT delivers higher doses to the tumour even considering a conservative constraint in the dose prescription to the normal tissue. Of note, in the PTV-LD BNCT has a dosimetric advantage with respect to carbon-ion therapy, especially because the minimum dose is about twice the dose delivered by carbon-ion therapy.

Normal tissue dose in Table 5 lists the mean and near-to-maximum dose delivered to normal brain and optic nerve for CIRT and BNCT. As in the former case, doses are converted into single fraction with BED formalism^27^ considering alpha/beta equal 2 Gy for Carbon-ion, while using isoeffective dose model for BNCT^8^. BNCT consistently delivers a lower maximum dose; the mean doses are comparable except for the left optic nerve where BNCT delivers a lower dose.

The effect of these dose distributions, is described by the TCP, which summarizes the therapeutic potential of the two types of radiation therapy. Table 6 lists the TCP of CIRT and BNCT calculated with the IT_STARTS toolkit. TCP of BNCT is higher than CIRT demonstrating that this option could be viable to treat the considered clinical case, applying the TCP model presented as a figure of merit for the benchmark.

**Table 4:** BNCT photon isoeffective dose values in the different volumes considered for the primary tumour, and single-fraction photon equivalent doses of the CIRT. Near-to-minimum refers to D98 and the near-to-maximum is D2.

BNCT	Near-to-Min	Dose to tumour ROIs	
		[Gy (IsoE)]	
		Mean	Near-to-Max
PTV-LD	21.6	27.5	32.9
PTV-HD	21.8	26.6	30.7
GTV	22.3	26.4	30.6

CIRT	Near-to-Min	Mean	Near-to-Max
PTV-LD	11.5	19.3	21.0
PTV-HD	17.5	20.5	21.1
GTV	19.1	20.7	21.3

**Table 5:** BNCT and CIRT single-fraction photon isoeffective mean and near-to-maximum dose values in the healthy brain and optic nerve.

BNCT	[Gy (IsoE)]	
	Mean	Near-to-Max
Brain	2.8	6.7
Right optic nerve	6.3	6.8
Left optic nerve	5.8	6.1

CIRT	Mean	Near-to-Max
Brain	0.2	10.9
Right optic nerve	5.0	12.2
Left optic nerve	7.6	10.8

**Table 6:** Comparison of TCPs ( Confidence Interval (CI) of 68%) obtained for the primary tumour treatment with carbon-ions and with BNCT.

	CIRT	BNCT
TCP (GTV)	0.31 [0.29, 0.34]	0.48 [0.46, 0.57]

### Recurrence

The second scenario considered the BNCT as the clinical option for the recurrence. The treatment planning was simulated without dose constraints from the first carbon ion irradiation. Dose was again limited to a maximum of 6 Gy in the mucosa, which determined a total treatment time of 26.9 min for each session.

**Figure 4:**
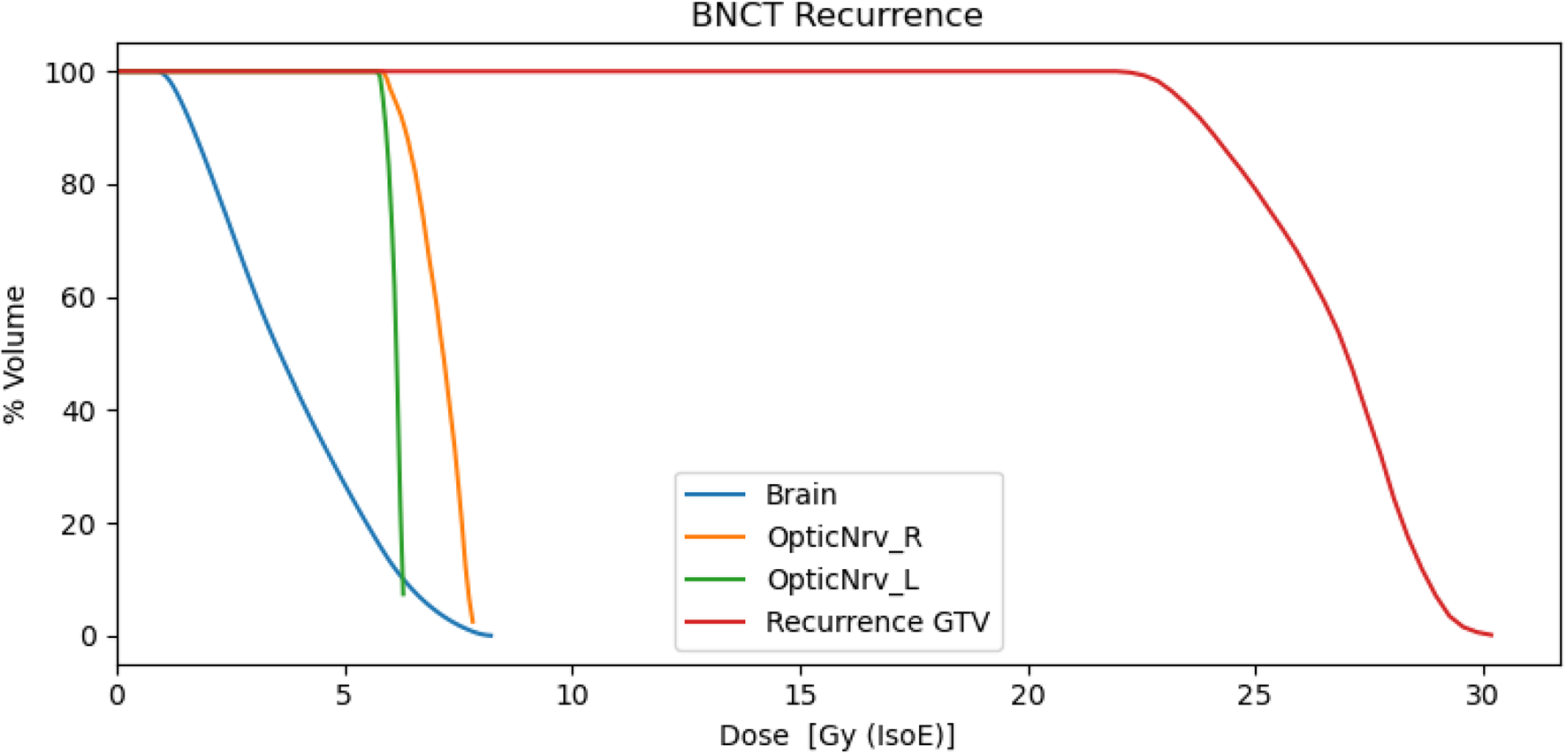
Dose Volume Histogram for the BNCT treatment of the recurrence and for the healthy organs.

The near-to-minimum, mean and near-to-maximum photon isoeffective BNCT doses to the recurrence and healthy tissues are reported in Table 7. The BNCT dosimetry for the recurrence is represented in the DVH plotted in Fig. 4.

**Table 7:** Photon isoeffective dose values of the BNCT treatment, calculated with the IT_STARTS toolkit, in the recurrence.

	BNCT		
	[Gy (IsoE; BNCT)]		
	Near-to-Min	Mean	Near-to-Max
Recurrence	22.9	27.0	29.0
Brain	1.2	3.6	7.5
Right optic nerve	6.0	7.2	7.8
Left optic nerve	5.8	6.1	6.3

The dose distribution obtained in the recurrence was used to calculate the TCP with the described method, giving a value of 0.54 [0.51, 0.57], higher than the TCP obtained for the treatment of GTV with CIRT (0.31). With the same consideration as before, this value of the TCP may suggest that BNCT could represent a therapeutic option for this recurrence, sparing the optic nerve, which receives a maximum dose lower than tolerance level.

**Figure 5:**
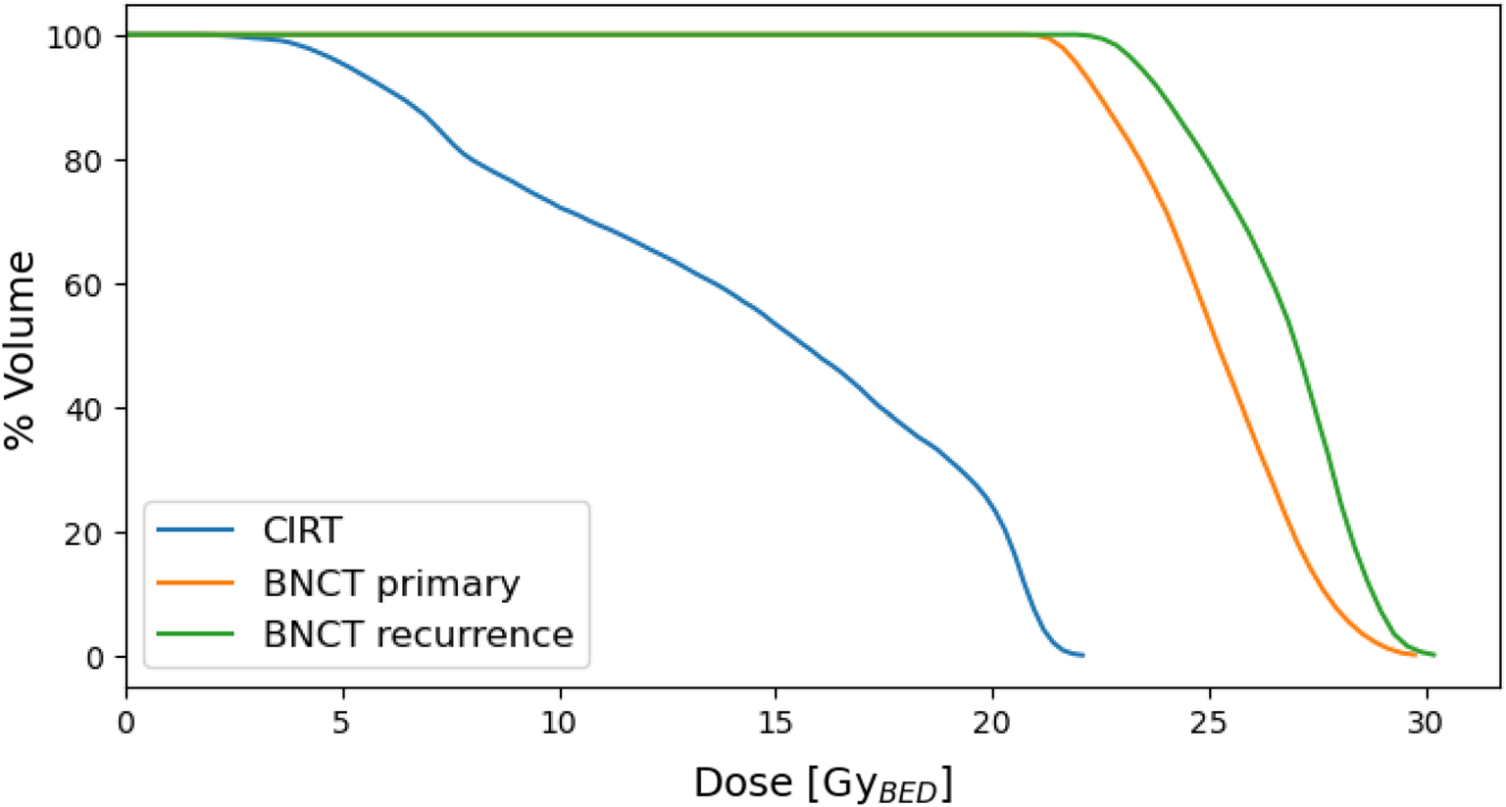
DVH for BNCT for the treatment of recurrence (green), DVH of the same recurrence in the BNCT treatment of the GTV (orange) and DVH for CIRT in the ROI corresponding to the recurrence (blue).

The fact that the volume of the recurrence was actually under-dosed in the CIRT, is shown in Fig. 5, which reports the comparison of the DVH obtained by BNCT for the treatment of recurrence and CIRT dosimetry in the ROI corresponding to the recurrence.

The treatment of the primary GTV would allow a dose coverage similar to the one that has been optimized for the treatment of recurrence, albeit with a lower minimum dose.

### Combination of BNCT and CIRT

At the time of the treatment, CIRT used constraints very conservative for the optic nerve, and today the dosimetry would be different^43^. However, even with the precision of CIRT, it is difficult to treat infiltrated targets with microscopic margins in contact with optic pathways. Due to its high LET, the irradiation would in fact cause irreversible damages both to tumour and healthy structures such as the optic nerve. On the other hand, BNCT offers a biological selectivity, depending on boron distribution, which targets tumour cells. These points highlight the advantages and disadvantages of the two treatment options. Charged particle therapy is capable of delivering a high and uniform dose in the tumour bulk volume. BNCT, albeit with limited penetration and uniformity, delivers high dose with biological selectivity, thus allowing hitting cancer cells, which may be latent and disseminated.

Table 8 reports the near-to-minimum, mean and near-to-maximum dose delivered to the GTV, PTV-LD and PT-HD in the combined treatment. Table 9 reports the dose delivered to optic nerves and normal brain.

**Figure 6:**
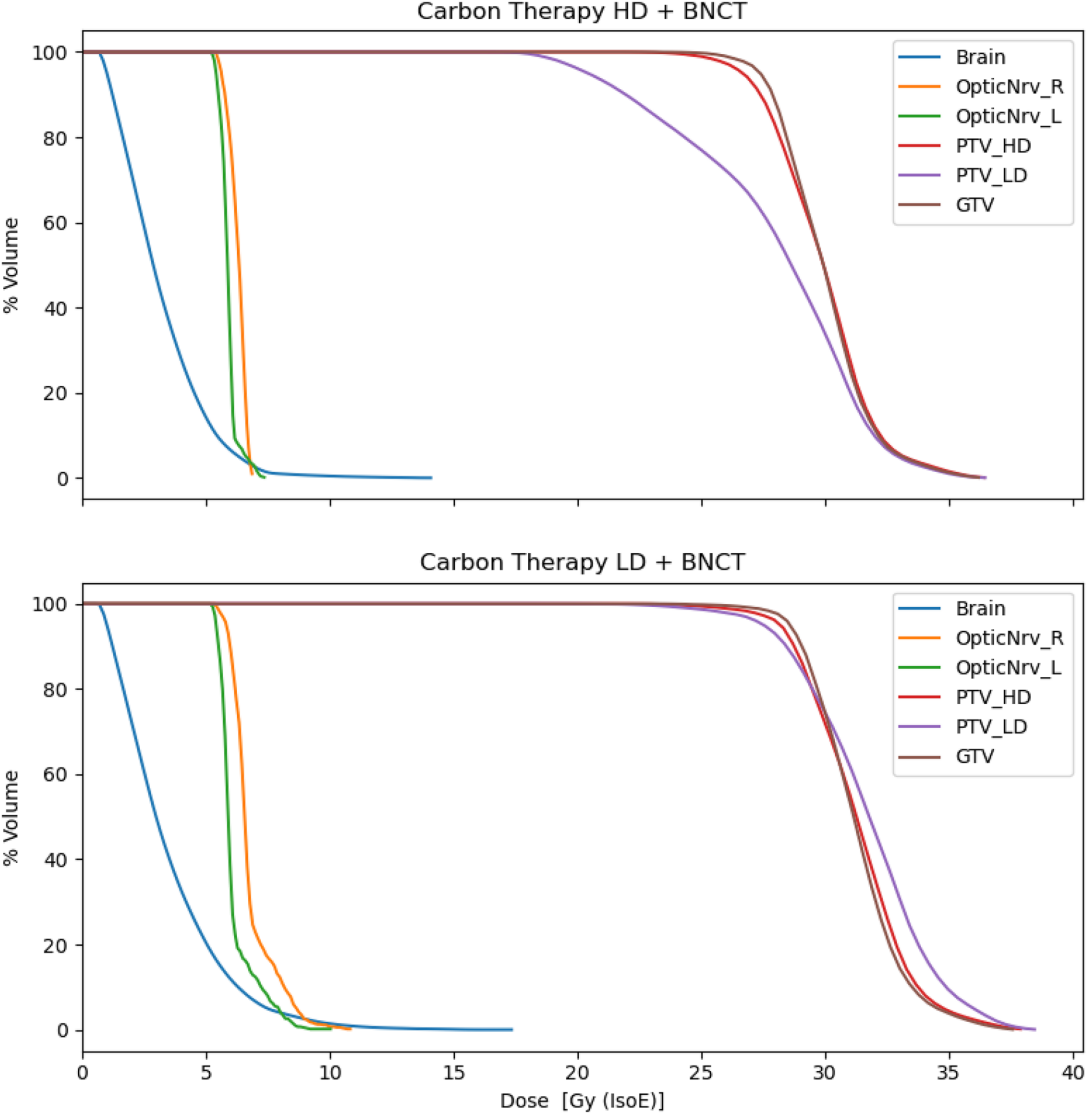
Top: Dose Volume Histograms (DVH) of the carbon ions treatment in the PTV-HD region combined with BNCT (doses in Gy (IsoE, BED) single-fraction). Bottom: Dose Volume Histograms of the carbon ions treatment in the PTV-LD region combined with BNCT (doses in Gy (IsoE, BED) single-fraction).

Table 10 reports the TCP evaluation for the two considered combinations. Combining BNCT with the CIRT of the PTV-LD is more advantageous with respect of the combination of BNCT and CIRT of the PTV-HD because the the dose in GTV is higher. This is also confirmed by TCP calculation, reported in Table 10. The value obtained for the combination of 1 BNCT session and the CIRT of the low-dose region leads to a value similar to the TCP of 2 sessions of BNCT. Comparing the DVH of the combined treatment (Fig. 6) with the DVH of BNCT alone shown in Fig. 3 it can be seen that the PTV-LD receives a more uniform dose. Considering that this was the region where the recurrence occurred, this dose distribution may be more effective in treating the cells dispersed outside the bulk.

**Table 8:** Combined CIRT and BNCT photon isoeffective dose values considering Carbon-ion treatment of the PTV-LD plus BNCT and Carbon-ion treatment of the PTV-HD plus BNCT.

	Tumour dose		
	[Gy (IsoE)]		
$^{12}$C LD + BNCT	Near-to-Min	Mean	Near-to-Max
GTV	27.9	31.1	35.8

$^{12}$C HD + BNCT	Near-to-Min	Mean	Near-to-Max
GTV	26.6	29.9	34.5

**Table 9:** Combined Carbon-ion and BNCT photon isoeffective dose values in the healthy brain and optic nerves.

	Healthy organs dose		
	[Gy (IsoE; BNCT)]		
$^{12}$C LD + BNCT	Near-to-Min	Mean	Near-to-Max
Brain	0.3	8.7	17.0
Right optic nerve	0.2	5.4	10.6
Left optic nerve	0.2	5.0	9.8

$^{12}$C HD + BNCT	Near-to-Min	Mean	Near-to-Max
Brain	0.3	7.0	13.8
Right optic nerve	0.1	3.1	6.2
Left optic nerve	0.1	3.7	7.2

**Table 10:** Comparison of TCPs (CI of 68%) obtained for the primary tumour treatment with carbon-ions and with BNCT.

	$^{12}$C LD + BNCT	$^{12}$C HD + BNCT
TCP (GTV)	0.52 [0.49, 0.55 ]	0.46 [0.43, 0.49]

## Discussion

**Figure 7:**
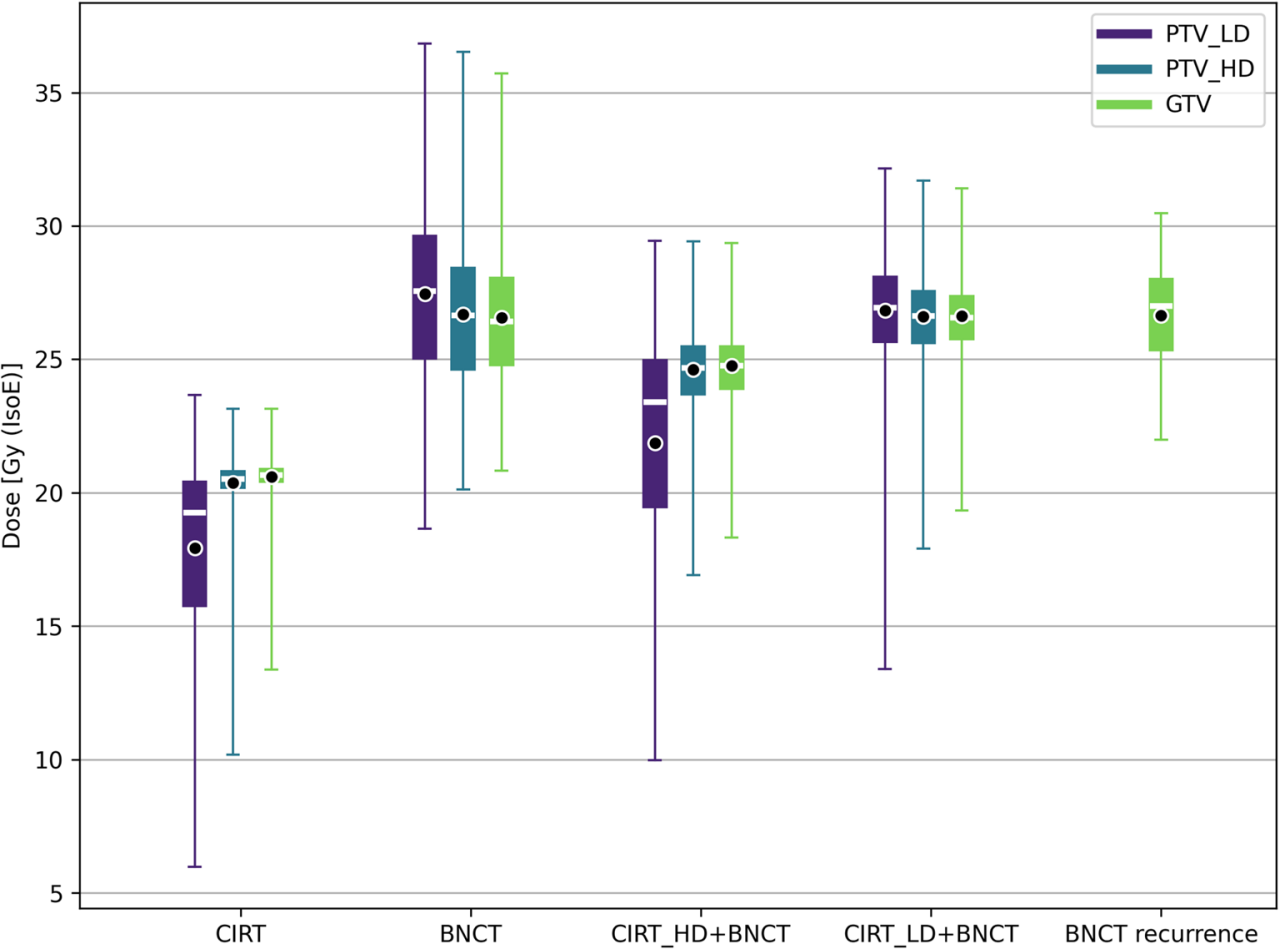
Dose distribution in tumour volumes for CIRT, BNCT and BNCT combined with carbon-ion for the treatment of the primary tumour. For comparison, also the tumour dose distribution for the treatment of the recurrence with BNCT alone is reported. The box extends from the first quartile to the third quartile of the data, with a white line at the median and a black dot at the mean.

**Figure 8:**
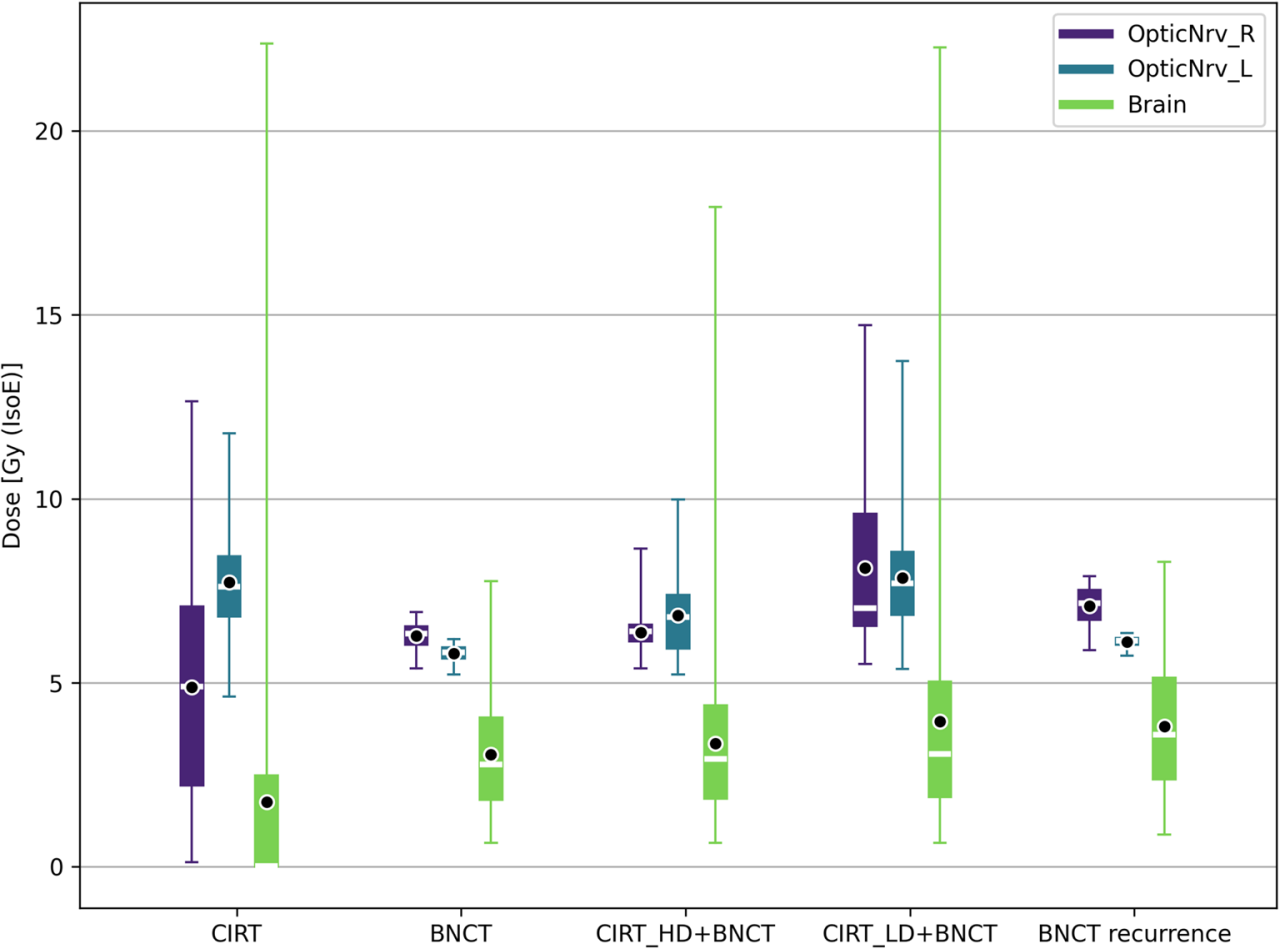
Dose distribution in healthy organs for CIRT, BNCT and BNCT combined with carbon-ion for the treatment of the primary tumour. For comparison, also the healthy tissue dose distribution for the treatment of the recurrence with BNCT alone is reported. The box extends from the first quartile to the third quartile of the data, with a white line at the median and a black dot at the mean.

Dose distributions to tumour and healthy tissues for the different tested treatments are summarized respectively in Figs. 7 and 8.

Figure 7 reveals that the dose distributions delivered with CIRT in the ROIs of the primary tumour are generally more uniform than those delivered with BNCT. However, the higher dose administered with BNCT would offer a higher TCP and a superior treatment of the possible infiltrated tumour cells in the PTV-LD and PTV-HD. It is worth considering that the dose in PTV-LD has been computed considering a uniform boron concentration, with a value typical of tumour. This leads to the minimum, mean and maximum dose values shown in this work. However, PTV-LD is healthy in the large majority of its volume, with possible infiltration of tumour: this dosimetry ensures that the latent nodules at least will absorb the minimum dose, listed in Table 4. The rest of the tissue, due to the differential boron concentration, would absorb a dose considerably lower. In fact, the near-to-maximum dose in the PTV-LD calculated with a boron concentration of 15 ppm is 8 Gy (IsoE), lower than the near-to-minimum dose absorbed by CIRT (see Table 4).

**Figure 9:**
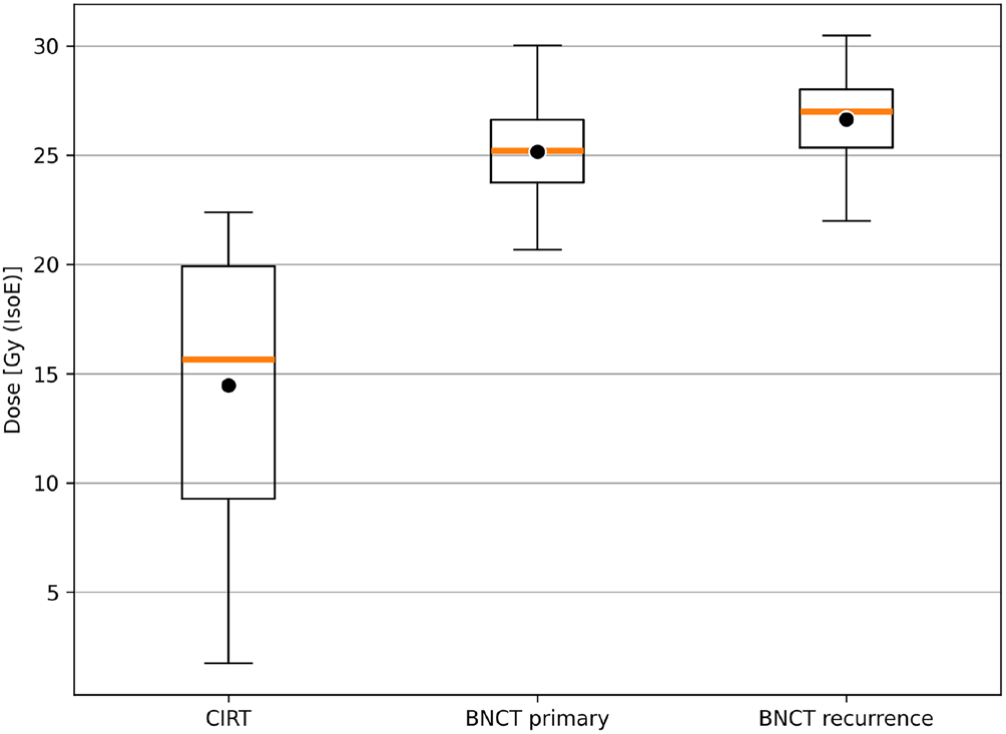
Dose distribution in the ROI corresponding to the recurrence volume for: CIRT, BNCT treatment of the primary tumour and BNCT treatment of the recurrence. The box extends from the first quartile to the third quartile of the data, with a yellow line at the median and a black dot at the mean.

Figure 9 gives an insight into the treatment of the recurrence. The box on the right represents the BNCT dosimetry of treatment conceived for the recurrence: the median dose delivered to the relapse is comparable to the one of BNCT in the GTV. This is reflected by a similar TCP. This suggests that BNCT may be able to control the tumour after its relapse in the optic nerve area, sparing the optic nerve. The box in the centre represents the dosimetry obtained in the treatment of the primary GTV superposed to the ROI volume of the recurrence, showing a similar outcome. This suggests that the treatment of the GTV also provides a dose coverage in the volume of recurrence possibly avoiding the relapse. Finally, the box on the left has been obtained with the dosimetry of CIRT superposed to the recurrence volume, showing that the relapse occurred in an under-dosed region.

In the final scenario where BNCT and carbon ion are combined, the dose distribution shows an advantage when one session of BNCT is added to the 9 fractions of carbon ion irradiation covering the PTV-LD compared to carbon-ion treatment alone. The minimum, mean and maximum dose value are higher in the tumour ROIs. This could represent an advantage compared to the original treatment in terms of TCP and treatment time, as patient would undergo only 9 fraction of CIRT and 1 session of BNCT, with a substantially higher TCP. The strategy of combination would ensure a uniform coverage of the GTV with charged particles also in the case that boron absorption would not be as uniform as assumed in the calculations.

For all these scenarios, the dose delivered to the healthy organs are comparable; the combined treatment (CIRT of PTV-LD plus BNCT) delivers a higher mean and maximum doses to the optic nerves, however still lower than the tolerance limit.

As BNCT would be available in Pavia at the same facility where CIRT is currently a therapeutic option, we worked in view of comparing and combining the two treatments. The investigation of this method is justified by the existence of extremely complex diseases, where CIRT might not fully cover the entire targeted volume. When combined with other particle therapies, the increased efficacy of BNCT owing to the availability of accelerators and, presumably, more effective boron carriers, will provide a therapeutic response for patients with more advanced tumors.

Head and neck tumors have shown excellent response to both BNCT and CIRT treatment. In this particular example, however, BNCT alone outperforms CIRT in terms of TCP and dosage values. The calculations were conducted assuming a constant boron concentration, as is the case with clinical BNCT. Nevertheless, CIRT coverage of the entire tumor would provide a more uniform dose distribution to regions of the GTV that might have taken less boron. Conversely, BNCT can more effectively target tumor regions which are closer to sensitive organs or infiltrated into the normal tissues.

## Conclusions

This work demonstrated in a clinical case that the photon isoeffective dose formalism proved suitable not only for comparing a proposed BNCT treatment with the original CIRT, but also evaluating a potential treatment combining the two treatment modalities. The results obtained show a possible procedure to evaluate the therapeutic potential of a BNCT treatment or the combination of BNCT with another form of hadrontherapy based on radiobiological figures of merit such as the TCP for non-uniform dose. Apart from the specific TCP values obtained in this clinical case, the objective of the work was to demonstrate the feasibility of such approach and explore for the first time the sum of dosimetry obtained with different radiation fields. The calculation of TCP using the traditional fixed RBE formalism in BNCT would lead to 100% in each case, which would be not realistic as already demonstrated in cited retrospective studies^5^. It is worth noting that all the assumptions made for the BNCT treatment planning simulations have been taken from a real BNCT clinical protocol in which about 100 patients have been treated. Moreover, the constraints have been kept as conservative as possible. About dosimetry in the GTV, it is important to stress that BNCT treatment planning prescribes the dose to the most radiosensitive tissue, and dose to the tumour is calculated knowing the boron concentration ratio. Future studies will consider possible issues in over-dosing the tumour in single irradiation sessions, which may cause toxicity to the rest of the body due to induced necrosis.

Combining the advantages of two types of particle therapy may enlarge the pool of patients accessing therapeutic options when no other strategies are available. However, further work is warranted to explore the clinical validity of the combination of these forms of radiotherapy. Therefore, future analyses using the methods described in this paper will include several clinical cases to produce adequate statistics.

## Data Availability

The datasets generated and analysed during the current study are not publicly available. Software for dosimetry calculation and analysis in simulations of treatment planning is available from the corresponding author on reasonable request.
